# Modeling of malaria vaccine effectiveness on disease burden and drug resistance in 42 African countries

**DOI:** 10.1038/s43856-023-00373-y

**Published:** 2023-10-13

**Authors:** Alisa Hamilton, Fardad Haghpanah, Mateusz Hasso-Agopsowicz, Isabel Frost, Gary Lin, Emily Schueller, Eili Klein, Ramanan Laxminarayan

**Affiliations:** 1 One Health Trust, Washington, D.C., USA; 2 World Health Organization, Geneva, Switzerland; 3 Imperial College London, London, UK; 4 Johns Hopkins University, Department of Emergency Medicine, Baltimore, MD USA; 5 One Health Trust, New Delhi, India; 6 Princeton University, Princeton, NJ USA; 7 University of Washington, Seattle, WA USA

**Keywords:** Malaria, Vaccines

## Abstract

**Background:**

The emergence of antimalarial drug resistance poses a major threat to effective malaria treatment and control. This study aims to inform policymakers and vaccine developers on the potential of an effective malaria vaccine in reducing drug-resistant infections.

**Methods:**

A compartmental model estimating cases, drug-resistant cases, and deaths averted from 2021 to 2030 with a vaccine against *Plasmodium falciparum* infection administered yearly to 1-year-olds in 42 African countries. Three vaccine efficacy (VE) scenarios and one scenario of rapidly increasing drug resistance are modeled.

**Results:**

When VE is constant at 40% for 4 years and then drops to 0%, 235.7 (Uncertainty Interval [UI] 187.8–305.9) cases per 1000 children, 0.6 (UI 0.4–1.0) resistant cases per 1000, and 0.6 (UI 0.5–0.9) deaths per 1000 are averted. When VE begins at 80% and drops 20 percentage points each year, 313.9 (UI 249.8–406.6) cases per 1000, 0.9 (UI 0.6–1.3) resistant cases per 1000, and 0.9 (UI 0.6–1.2) deaths per 1000 are averted. When VE remains 40% for 10 years, 384.7 (UI 311.7–496.5) cases per 1000, 1.0 (0.7–1.6) resistant cases per 1000, and 1.1 (UI 0.8–1.5) deaths per 1000 are averted. Assuming an effective vaccine and an increase in current levels of drug resistance to 80% by 2030, 10.4 (UI 7.3–15.8) resistant cases per 1000 children are averted.

**Conclusions:**

Widespread deployment of a malaria vaccine could substantially reduce health burden in Africa. Maintaining VE longer may be more impactful than a higher initial VE that falls rapidly.

## Plain language summary

Malaria can become resistant to the drugs used to treat it, posing a major threat to malaria treatment and control. An effective vaccine has the potential to reduce both resistant infections and antimalarial drug use. However, how successfully a vaccine can protect against infection (vaccine efficacy) and the impact of increasing drug resistance remain unclear. Using a mathematical model, we estimate the impact of malaria vaccination in 42 African countries over a 10-year period in multiple scenarios with differing vaccine efficacy and drug resistance. Our model suggests that a moderately effective vaccine with sustained protection over a long period could avert more resistant infections and deaths than a vaccine that is highly protective initially but lowers in efficacy over time. Nevertheless, implementation of an effective malaria vaccine should be accelerated to mitigate the health and economic burden of drug resistance.

## Abstract (web summary)

Hamilton et al. model the impact of a malaria vaccine on malaria cases, drug resistance, and deaths if administered yearly to infants in 42 African countries. They find that even a moderately effective vaccine could substantially reduce malaria burden, with a vaccine that maintains its efficacy over time being most impactful.

## Introduction

In 2021, there were an estimated 619,000 deaths globally from malaria, approximately 80% of which were in children under five^1^. Ninety-six percent of these deaths occurred in sub-Saharan Africa^1^. Global gains in malaria control from antimalarials and vector control products (e.g., insecticide-treated bed nets and indoor residual spraying) have plateaued in recent years, and disruptions due to the COVID-19 pandemic have hindered country efforts to prioritize and implement interventions^2^. Of particular concern to malaria elimination efforts is the spread of partial resistance to artemisinin combination therapies (ACTs), the recommended first-line treatment for *Plasmodium falciparum* in sub-Saharan Africa^3–5^. ACTs consist of artemisinin derivatives that clear most infections within three days of treatment, while partner drugs are long-acting and clear any remaining parasitemia^6^. Already widespread in South East Asia, artemisinin partial resistance has now been confirmed in Eritrea, Rwanda, and Uganda^2,7,8^, and molecular mutations associated with resistance (*Pfkelch13*) have been detected in several other African countries^9^. Artemisinin partial resistance results in delayed parasite clearance while resistance to partner drugs in ACTs is associated with recrudescence^6,10–12^. Both mechanisms lengthen the duration of illness and prolong periods of transmission, thus increasing health burden, health system costs, and productivity loss.

Projections by Laxminarayan et al. suggest that without interventions to slow its spread, ACT resistance could spread as quickly as chloroquine resistance in the late 20th century^13^, outpacing pharmaceutical development and reducing the effectiveness of current treatments^14^. Chloroquine resistance, first emerged in South East Asia and Latin America in the 1960s and in East Africa in the 1970s^15,16^ and quickly spread to other malaria endemic countries in sub-Saharan Africa^5,15^. In the 1980s, hospital studies from Nigeria, Congo, and the Democratic Republic of the Congo reported a two to three-fold increase in malaria deaths and admissions for severe malaria^17^. Population-based studies from Senegal suggested malaria mortality among children increased nearly six-fold in areas that had previously achieved low levels of mortality using chloroquine for prophylaxis and treatment^17,18^. Rapid spread of ACT resistance would have dire health and economic consequences. A modeling study of artemisinin resistance in all malaria endemic countries estimated $385 million USD in annual resistance-related productivity losses and an additional $32 million USD in annual medical expenditures^19^.

Vaccines are a powerful tool against the emergence and spread of antimicrobial resistance (AMR). By reducing both drug-resistant and sensitive infections^14,20–22^, vaccines reduce reliance on drug therapies and selective pressure driving resistance, averting significant health and economic burden^14^. The World Health Organization has recognized the role of vaccines in combatting AMR and has developed a strategy to optimize the use of vaccines for this purpose^14,20,21,23–26^. Recent studies by ARVac, a consortium of universities and organizations modeling the impact of vaccines on AMR, have quantified estimated health burden averted for several vaccines. A future rifampicin-resistant tuberculosis (RR-TB) vaccine could avert ~10% of resistant cases and ~7.3% of deaths from 2020 to 2035 in the 30 countries that account for 90% of global RR-TB incidence^27^. Pneumococcal conjugate vaccines led to absolute reductions in the proportions of pneumococci resistant to penicillin, sulfamethoxazole-trimethoprim, and third-generation cephalosporins by an estimated 7.3%, 16%, and 4.5%, respectively, on average across all regions during the 10-year period after their introduction^23^. Under current coverage levels, rotavirus vaccines prevent an estimated 13.6 million episodes of antibiotic-treated illness among children under 5 years old in low- and middle-income countries, corresponding to a reduction of ~11.4% in the first 2 years of life compared to a situation without a vaccine^20^.

Developing a vaccine against a parasite like malaria is difficult, however, due to the complexity of the malaria parasite life cycle and our incomplete understanding of the immune system’s response^28^. The only candidate recommended by the WHO, RTS,S/AS01, took over 30 years to gain regulatory approval and enter pilot implementation studies. RTS,S/AS01 is a pre-erythrocytic vaccine that targets the hepatic stage of *P. falciparum*, specifically the circumsporozoite protein, triggering the immune system to create anti-circumsporozoite antibodies. In a large multi-site Phase III trial, RTS,S/AS01 demonstrated ~50% efficacy against clinical or severe malaria during 12 months follow-up and ~36% efficacy during a median of 48 months follow-up. Higher efficacy was achieved using a seasonal delivery strategy, reaching vaccine efficacy that was non-inferior to that provided by seasonal malaria chemoprevention (which reduces ~75% of malaria cases)^29^. In large WHO-coordinated pilot implementations in which the vaccine was provided through routine immunization systems in Ghana, Malawi, and Kenya, introduction of the vaccine resulted in a ~30% reduction in hospitalized severe malaria^30^. A second pre-erythrocytic malaria vaccine R21/Matrix-M, an adaptation of RTS,S/AS01, is in ongoing Phase III trials. R21 Matrix-M uses the same circumsporozoite protein antigen as RTS,S/AS01, but at a higher concentration, as well as the Matrix-M adjuvant used in the Novavax COVID-19 vaccine^31^, which stimulates humoral and cellular immune responses to vaccines, similar to AS01^32^. If recommended for use, R21/Matrix-M could improve the supply for malaria vaccines to help reach a larger number of children at risk of clinical malaria^31^. Similar to the higher efficacy observed when RTS,S/AS01 was delivered just before peak season in areas of highly seasonal malaria transmission, R21, in a Phase IIb trial demonstrated ~77% efficacy over 12 months follow-up^31,33^. Seasonal administration of RTS,S/AS01 has also shown similar levels of protective efficacy, and with added impact when combined with chemoprevention^29^.

The health and economic impacts of malaria vaccines, including RTS,S/AS01, have been simulated by several mathematical models of varying complexity^34^. Deterministic compartmental models have been used to estimate the impact of vaccination on malaria transmission^35,36^, and stochastic individual-based models have been employed to project vaccine-averted morbidity and mortality^37–39^. In a comparison of four models^37–40^, Penny et al. concluded that RTS,S/AS01 could avert a median of 116,480 (range 31,450–160,410) clinical cases and 484 (189–859) deaths per 100,000 fully vaccinated children with a four-dose schedule in Sub-Saharan Africa over a 15-year time horizon^41^. Hogan et al. estimated 4.3 million malaria cases and 22,000 deaths in children under 5 years old could be averted annually assuming the same vaccine coverage as Diphtheria tetanus toxoid and pertussis vaccination (DTP3), country-level prioritization in high endemicity areas, limited to 30 million doses per year. None of these models examined the impact of a vaccine on drug-resistant cases.

In this study, we use a metapopulation model to approximate cases, drug-resistant cases, and deaths averted with a malaria vaccine administered yearly to 1-year-olds in 42 African countries under different VE scenarios. Health events averted are tracked for each cohort of infants entering the 10-year period from 2021 to 2030. The goal of the study is to provide aggregate projections of burden averted for the WHO Africa Region. Our results indicate that even a moderately effective vaccine could significantly reduce the burden of malaria and antimalarial drug resistance, making vaccines an important tool alongside other prevention and control strategies.

## Methods

### Model and analysis

We constructed a deterministic compartmental model with a 1-year time step to approximate the effectiveness of widespread deployment of a malaria vaccine under three VE scenarios and a fourth scenario of increasing resistance (Table 1). We defined VE as efficacy against clinical malaria caused by *P. falciparum*. In Scenario 1, VE remained constant at 40% for 4 years and dropped to 0% in year five. In Scenario 2, VE began at 80% and dropped 20 percentage points each year until reaching 0% in year five. The second scenario approximated results of VE from the Phase 3 trial of seasonal RTS,S vaccination or the Phase IIb trial of seasonal R21/Matrix-M vaccination in areas of highly seasonal transmission (i.e., the majority of malaria episodes occur within a consecutive 4- or 5-month period), which showed an average of 75% during 12 months^33^. As with many vaccines, R21/Matrix-M (and RTS,S/AS01) efficacy is likely to wane with time; we approximated waning using an incremental decrease in VE in Scenario 2 as our model used a 1-year time step. In Scenario 3, VE remained constant at 40% for the entire study period of 10 years. Given the complexity of factors impacting VE, it is difficult to generalize exactly how efficacy will change over time and at scale; thus, we included Scenario 3 as a comparator. To account for a situation of rapidly increasing resistance, such as with chloroquine in the 1980s and 90s^17^, we modeled a fourth “worst-case” scenario using the same VE from Scenario 1 with resistance to ACTs increasing linearly to 80% by 2030 (Table 1).

**Table 1:** Vaccine efficacy (VE) and drug resistance by year for each scenario modeled

	Baseline		Scenario 1		Scenario 2		Scenario 3		Scenario 4 (rapidly increasing drug resistance)	
Year	VE (%)	Resistance	VE (%)	Resistance	VE (%)	Resistance	VE (%)	Resistance	VE (%)	Resistance
2021	0	Country-specific (constant)	40	Country-specific (constant)	80	Country-specific (constant)	40	Country-specific (constant)	40	Country-specific (increases linearly to 80% by year 2030)
2022	0		40		60		40		40	
2023	0		40		40		40		40	
2024	0		40		20		40		40	
2025	0		0		0		40		0	
2026	0		0		0		40		0	
2027	0		0		0		40		0	
2028	0		0		0		40		0	
2029	0		0		0		40		0	
2030	0		0		0		40		0	

In the baseline scenario, VE remains 0% and drug resistance remains constant for each year of the study period. In Scenario 1, VE is 40% for the first 4 years and drops to 0% in year. In Scenario 2, VE begins at 80% and drops 20 percentage points each year until reaching 0% in year five. In Scenario 3, VE remains constant at 40% for each study year. Country-specific delayed parasite clearance rates (DPCs) constructed from therapeutic efficacy studies were used as proxies for drug resistance. Drug resistance remained constant for each scenario except Scenario 4, which modeled a worst-case scenario where each countries DPC reached 80% in year 2030.

**Fig. 1:**
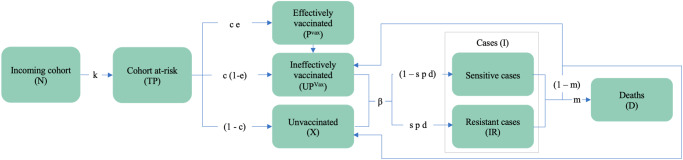
Malaria model diagram. One-year-olds at risk of malaria enter one of three compartments (effectively vaccinated, ineffectively vaccinated, or unvaccinated). In scenarios where vaccine efficacy (VE) changes by year, a proportion of effectively vaccinated individuals lose immunity and enter the ineffectively vaccinated state. A proportion of those without an effective vaccine move to one of two infected compartments (sensitive cases and resistant cases). A proportion of combined cases (both sensitive and resistant) lead to death each year, and the remainder return to either the ineffectively vaccinated or unvaccinated state, respectively. A ‘case’ is defined as an episode of symptomatic malaria, and multiple infections per year in one child are assumed to be captured by annual incidence and prevalence rates. Only incoming infants are vaccinated without a proposed catch-up campaign. The model is run for each cohort entering the study period, for each year, and for each country with results summed to generate cumulative estimates. Parameter definitions and sources are provided in Supplementary Table 1, and country-specific values are provided in Supplementary Data 1.

In the Phase III trial, RTS,S/AS01 was given as a 3-dose primary series starting at ages 5–17 months at first dose, with a 4-week interval between doses, followed by a fourth booster dose 18 months after the third dose. The vaccine protection was observed after three doses, and it was prolonged by the fourth dose^42^. Because our model used a 1-year time step, we approximated a multi-dose schedule by assuming vaccinated children receive all three doses in their first year of life with immunity beginning after effective vaccination in the second year of life. In reality, the fourth dose would be given in the second year of life to ensure lengthy protection. We used a discrete model of patient infection states, in which we assumed a new cohort of 1-year-olds is introduced each year and followed over the course of 10 years (Fig. 1). Children enter the model as effectively vaccinated, ineffectively vaccinated, or unvaccinated. “Effectively vaccinated” refers to children who develop immunity after vaccination, while “ineffectively vaccinated” refers to children who do not develop immunity after vaccination. Children without an effective vaccine (i.e., those who are unvaccinated or ineffectively vaccinated) become infected with a drug-sensitive or drug-resistant parasite according to age-specific incidence rates. After vaccinated individuals lose immunity due to waning, they enter the ineffectively vaccinated state. A proportion of clinical cases (both sensitive and resistant) lead to death each year according to country-specific case fatality rates (CFRs), and the remainder return to either ineffectively vaccinated or unvaccinated states, respectively, allowing for reinfection in subsequent years. We assumed only incoming 1-year-olds are vaccinated to approximate a routine vaccination schedule; a catch-up campaign was not modeled.

First, we estimated the number of cases, drug-resistant cases, and deaths in a baseline scenario without a vaccine. The target population (*TP*) for each cohort (*ct*) was calculated by multiplying the number of incoming 1-year-olds (*N*_*ct*_) by the proportion of the population “at risk” (*k*) in each country.

1 $$T{P}_{{ct}}={N}_{{ct}}{k}$$

The target population was then multiplied by the coverage rate (*c*) and the vaccine efficacy rate in year *y* (*e*_*y*_) to calculate the number of children effectively vaccinated (${P}_{y}^{{Vax}}$ for “protected vaccinated”) in that year.

2 $${P}_{y}^{{Vax}}=T{P}_{{ct}}c{e}_{y}$$

The number of ineffectively vaccinated children (${{UP}}_{y}^{{Vax}}$ for “unprotected vaccinated”) was calculated by subtracting effectively vaccinated children from all vaccinated children and the number of deaths (*D*) from the previous years.

3 $${{UP}}_{y}^{{Vax}}=T{P}_{{ct}}c-{P}_{y}^{{Vax}}-\mathop{\sum }\limits_{j=2021}^{y-1}{D}_{j}^{{Vax}}$$

Cases were calculated by multiplying the number of ineffectively vaccinated children and the number of unvaccinated children (*X*_*y*_) by age-specific incidence rates (*β*_*y*_) for each country.

4 $${I}_{y}^{{Vax}}=U{P}_{y}^{{Vax}}{\beta }_{y}$$

5 $${I}_{y}^{{NoVax}}={X}_{y}{\beta }_{y}$$

ACT-resistant cases (*IR*_*y*_ for “infections resistant”) were estimated by applying country-specific treatment received rates (*r*) and delayed parasite clearance rates (*d*) to cases.

6 $$I{R}_{y}=\left({I}_{y}^{{Vax}}+{I}_{y}^{{NoVax}}\right){rd}$$

Deaths were estimated by applying country-specific case fatality rates (*m*) to the number of ineffectively vaccinated and unvaccinated children, which included both sensitive and resistant cases.

7 $${D}_{y}^{{Vax}}={I}_{y}^{{Vax}}m$$

8 $${D}_{y}^{{NoVax}}={I}_{y}^{{NoVax}}m$$

Next, we calculated all outcomes for each VE scenario by changing the value of *e*_*y*_ (vaccine efficacy [VE]) depending on the year (Table 1). The model was run for each cohort, for each year, and for each country for the 10-year period with results summed to generate cumulative estimates for each outcome. Burden averted was estimated by comparing outcomes of the respective scenarios with the baseline scenario after aggregating at the country-year level.

Uncertainty intervals (UI) were calculated using Monte Carlo (MC) simulations with 1000 iterations, assuming triangular distributions. A range of ±10% was applied to population estimates and a range of ±20% was applied separately to treatment received, delayed parasite clearance, treatment failure, and mortality rates. The Global Health Observatory provided lower and upper bounds for the estimated number of annual malaria cases, and these ranges were used in the MC simulations. Parameter values, definitions, and sources are detailed in Supplementary Table 1. Data were processed using Microsoft Office Excel 16.51, Stata 16, and Python 3.8. The final analyses and MC simulations were conducted in Python 3.8 with visualizations generated in RStudio 1.3.

### Data sources

Population data were obtained from the United Nations World Population Prospects, which account for population growth and provide the estimated number of people in thousands for each year of life (0–100) by country-year^43^. We used the number of incoming 1-year-olds for each year of the study to approximate a vaccine administered to children aged 5–17 months. We used country-specific coverage rates for DTP3 vaccination of 1-year-olds as a proxy for vaccine coverage^44^. A ‘case’ was defined as an episode of symptomatic malaria and based on annual estimates reported by the WHO and the University of Washington’s Institute for Health Metrics and Evaluation (IHME). Age-stratified incidence rates (annual cases per 1000 population) were constructed for children under five and children over five by applying the proportion of cases attributed to each age group from IHME^45^ to the number of estimated annual cases from the WHO^46^ for each country (Supplementary Methods). These estimates include instances of multiple cases experienced by one child per year. We assumed cases reported by the WHO and IHME were all caused by *P. falciparum*, which is the dominant malaria species in Africa. A country’s proportion of the population “at risk” (i.e., number of people living in malarial areas) was taken from the World Malaria Report^2^. Supplementary Table 1 details model parameter definitions and sources.

Country-specific values for the percentage of children under five with fever receiving anti-malarial drugs were used as a proxy for treatment received rates (TRRs). The most recent year’s data for each country were acquired from the World Bank and were originally reported by UNICEF, the State of the World’s Children, Childinfo, and Demographic and Health Surveys^47^. Country-specific delayed parasite clearance rates (DPCs), defined as the proportion of patients with parasitemia on day three of treatment, were used as a proxy for resistance rates as suggested by the WHO^48^. DPCs were estimated using data from therapeutic efficacy studies occurring between 2010–2020 downloaded from the Malaria Threat Map^9^. These studies report DPCs for all ACT drugs (AL, AS + SP, AS-AQ, AS-MQ, AS-PY, or DHA-PPQ;). Drug names are detailed in Supplementary Table 2, and country-specific rates are provided in Supplementary Data 1. Rates from studies for *P. falciparum* with 30 or more samples were averaged across all years to construct a rate for each country. Values for countries with missing TRRs (*N* = 1) and missing DPCs (*N* = 7) were imputed using Bayesian multivariate regression with GDP per capita and under-five mortality per 1000 population as predictor variables (Supplementary Table 3; Supplementary Figure 1). Variables were selected as proxies for the strength of a country’s economy and healthcare system, which can be indicative of malaria treatment and surveillance capacity. We used the same method to calculate treatment failure rates (TFRs), which likely overestimate the role of resistance, and were used instead of DPCs in a sensitivity analysis, providing an upper bound for scenarios where drug resistance remains constant over time (Supplementary Table 4; Supplementary Figure 2). The WHO defines treatment failure as the development of severe malaria symptoms within three days of treatment (early treatment failure), parasitemia in patients with signs of early treatment failure or fever between day four and the end of follow-up, or parasitemia between day seven and the end of follow-up (late treatment failure)^49^.

Case fatality rate (CFR) was defined as the proportion of cases resulting in death^2^. A CFR of 0.256% was used for low-transmission countries (Botswana, Comoros, Eritrea, Eswatini, Ethiopia, Madagascar, Namibia, Zimbabwe) following WHO methodology^2^. For the remaining high-transmission countries, we constructed country-specific CFRs by dividing estimated total malaria deaths by estimated total malaria cases, assuming all cases and deaths are caused by *P. falciparum* in these countries. Assuming countries with CFRs less than 0.256% using this approach were low-transmission countries (*N* = 18), we replaced these rates with 0.256% to be consistent with WHO methodology (Supplementary Data 1). The most recent total case and death data for malaria were obtained from the WHO Global Health Observatory for this calculation^50,51^. Parameter definitions, values, and sources are detailed in Supplementary Table 1 (model parameters), Supplementary Data 1 (country-specific values), and Supplementary Data 2 (model input data). As all data was publicly available and de-identified; ethics committee approval was not required for this study. Data sources and analysis code are publicly available on GitHub^52^.

### Reporting summary

Further information on research design is available in the Nature Portfolio Reporting Summary linked to this article.

## Results

**Fig. 2:**
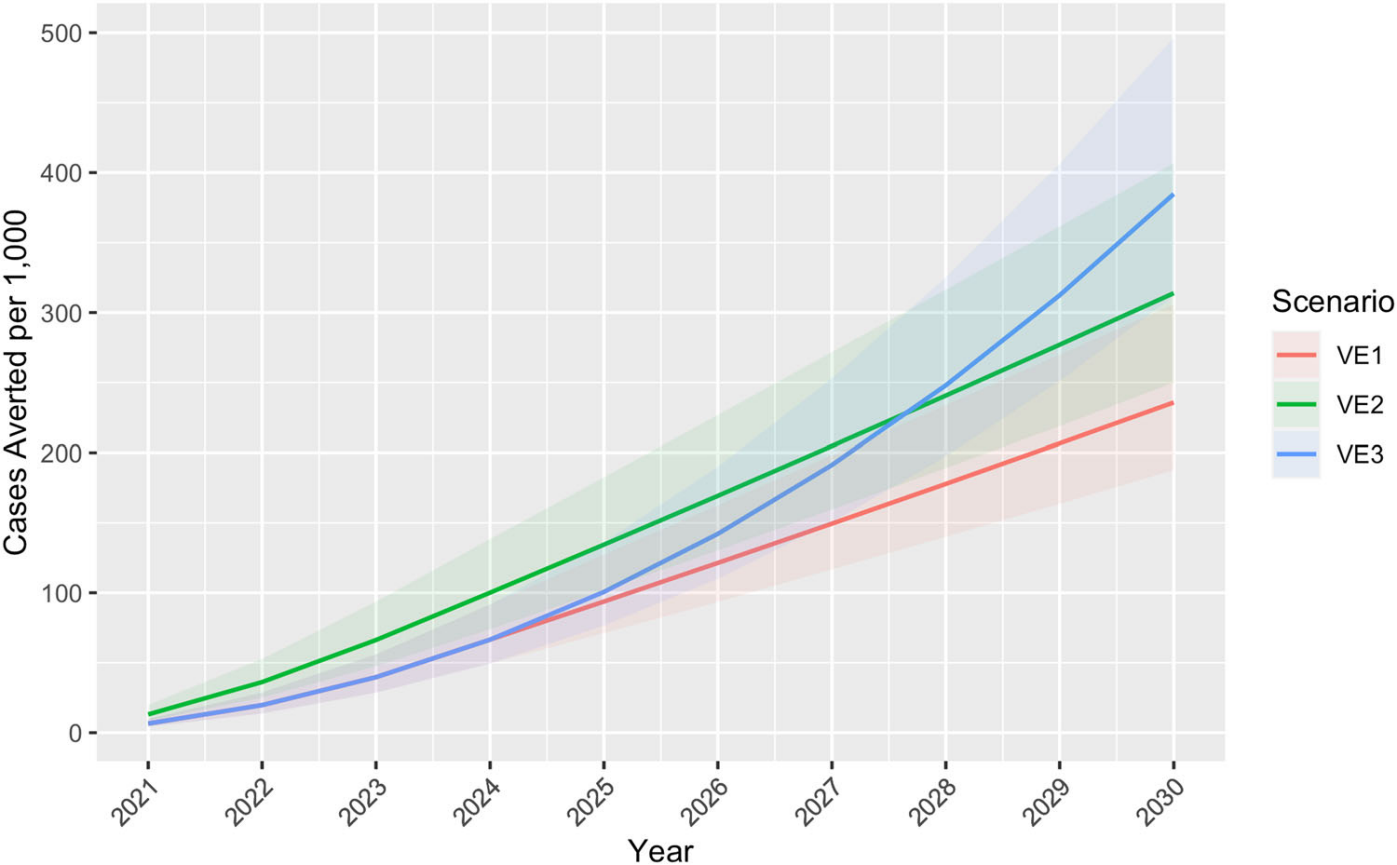
Cumulative cases averted per 1000 children by vaccine efficacy scenario, WHO Africa Region 2021–2030. Under Scenario 1 in which vaccine efficacy (VE) remained a constant 40% for 4 years and dropped to 0% in year five, 235.7 (Uncertainty Interval [UI] 187.8–305.9) cases per 1000 children were projected to be averted. Scenario 2, in which VE began at 80% and dropped 20 percentage points each year until reaching 0% in year 5, resulted in 313.9 (UI 249.8–406.6) cases averted per 1000 children. Scenario 3, in which VE remained a constant 40% for the entire study, resulted in approximately 384.7 (UI 311.7–496.5) cases averted per 1000 children.

**Fig. 3:**
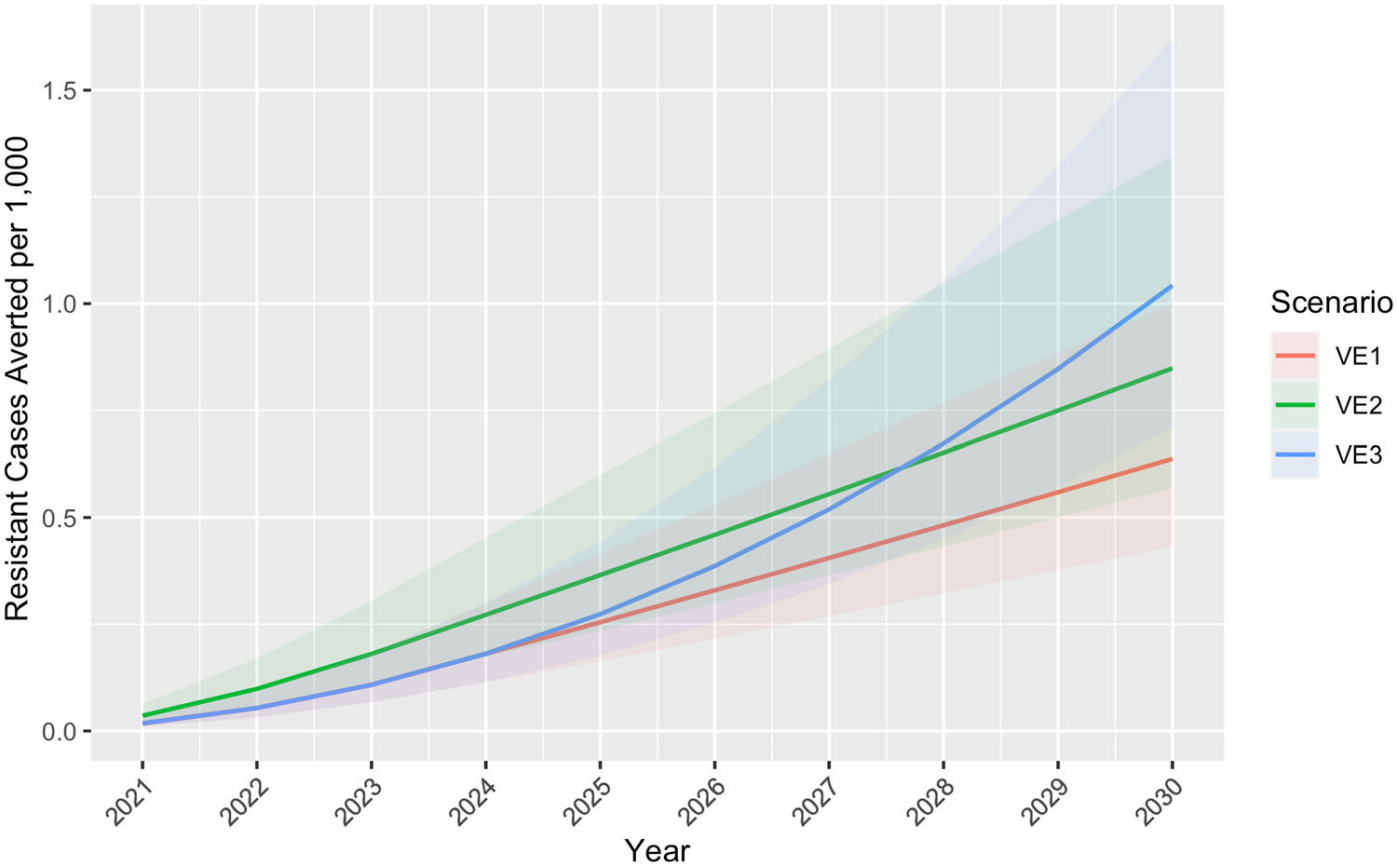
Cumulative resistant cases averted per 1000 children by vaccine efficacy scenario, WHO Africa Region 2021–2030. Under Scenario 1 in which vaccine efficacy (VE) remained a constant 40% for 4 years and dropped to 0% in year five, 0.6 (Uncertainty Interval [UI] 0.4–1.0) resistant cases per 1000 children were projected to be averted. Scenario 2, in which VE began at 80% and dropped 20 percentage points each year until reaching 0% in year 5, resulted in 0.8 (UI 0.6–1.3) resistant cases averted per 1000 children. Scenario 3, in which VE remained a constant 40% for the entire study, resulted in approximately 1.0 (UI 0.7–1.6) resistant cases averted per 1000 children.

**Fig. 4:**
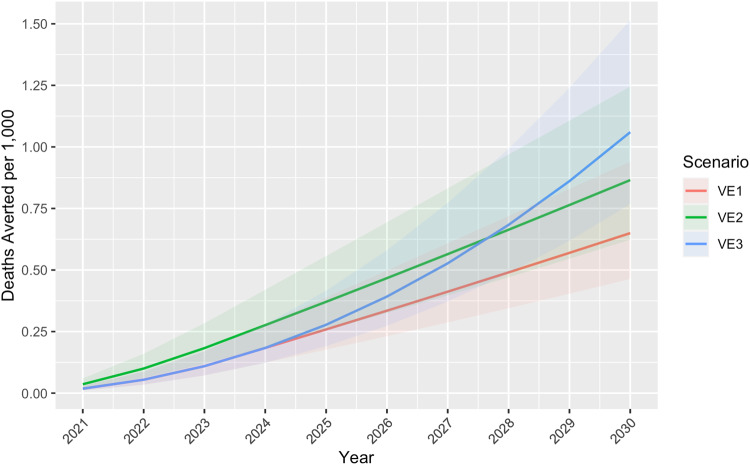
Cumulative deaths averted per 1000 children by vaccine efficacy scenario, WHO Africa Region 2021–2030. Under Scenario 1 in which vaccine efficacy (VE) remained a constant 40% for 4 years and dropped to 0% in year five, 0.6 (Uncertainty Interval [UI] 0.5–0.9) deaths per 1000 children were projected to be averted. Scenario 2, in which VE began at 80% and dropped 20 percentage points each year until reaching 0% in year 5, resulted in 0.9 (UI 0.6–1.2) deaths averted per 1000 children. Scenario 3, in which VE remained a constant 40% for the entire study, resulted in approximately 1.1 (UI 0.8–1.5) deaths averted per 1000 children.

**Fig. 5:**
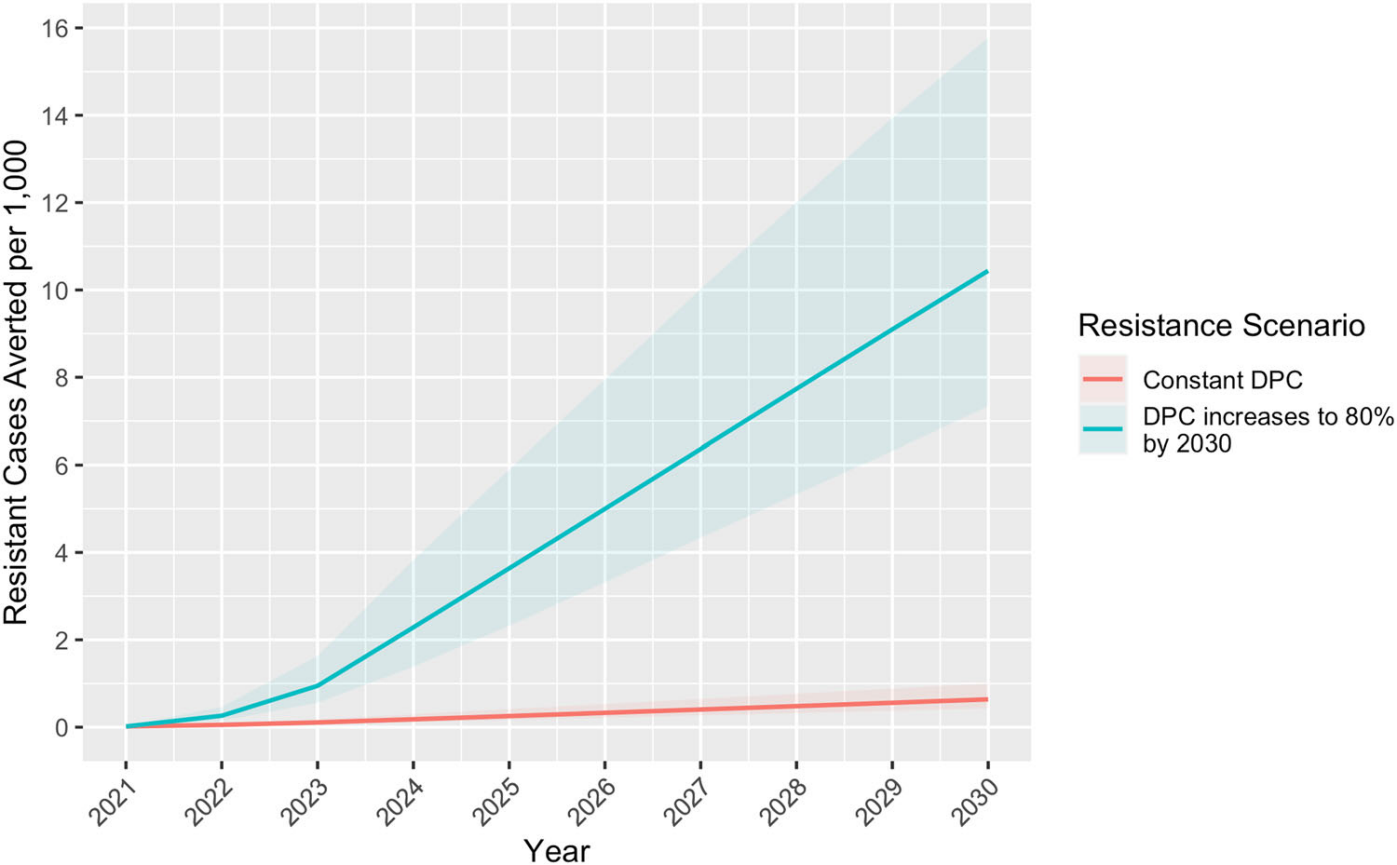
Cumulative resistant cases averted per 1000 children under two drug resistance scenarios. Given a vaccine with 40% efficacy and 4 years’ duration (Scenario 1), approximately 0.6 (Uncertainty Interval [UI] 0.4–1.0) cases per 1000 children were projected to be averted if delayed parasite clearance rates (DPCs) remained constant over the 10-year study period. If drug resistance developed rapidly and DPCs reached 80% in ten years, 10.4 (UI 7.3–15.8) resistant cases per 1000 children were projected to be averted with an effective vaccine and VE waning the same as Scenario 1.

Under the baseline scenario without a vaccine, we projected 1409.1 (UI 1143.6–1808.7) malaria cases per 1000 children, of which 3.8 (UI 2.6–5.9) per 1000 were ACT-resistant, resulting in 3.8 (UI 2.8–5.5) deaths per 1000 among cases with both sensitive and resistant parasites (Table 2; Supplementary Figures 3–5). These results are cumulative over the 10-year period 2021–2030 for the WHO Africa Region, which included 42 countries in our study. Under Scenario 1 in which vaccine efficacy remained constant at 40% for 4 years and dropped to 0% in year five, 235.7 (UI 187.8–305.9) cases per 1000, 0.6 (UI 0.4–1.0) resistant cases per 1000, and 0.6 (UI 0.5–0.9) deaths per 1000 were averted, corresponding to a reduction of ~17% in all outcomes compared to baseline (Table 2; Figs. 2–4). Scenario 2, in which VE began at 80% and dropped 20 percentage points each year until reaching 0% in year five, resulted in 313.9 (UI 249.8–406.6) cases per 1000, 0.9 (UI 0.6–1.3) resistant cases per 1000, and 0.9 (UI 0.6–1.2) deaths per 1000 averted, corresponding to a reduction of ~22% in all outcomes compared to baseline. Scenario 3, in which VE remained constant at 40% for the entire study, resulted in 384.72 (UI 311.70–496.47) cases per 1000, 1.0 (UI 0.7–1.6) resistant cases per 1000, and 1.1 (UI 0.8–1.5) deaths per 1000 averted, corresponding to a reduction of ~27% in all outcomes compared to baseline. When resistance ACT increased rapidly assuming the same VE as Scenario 1 but with DPCs increasing to 80% by 2030, the same number of cases and deaths were projected to be averted as Scenario 1, but more resistant cases per 1000 (10.4 [UI 7.3–15.8] vs 0.64 [UI 0.4–1.0]) were projected to be averted (Table 2; Fig. 5). Using TFRs instead of DPCs, 2.0 (1.3–3.1) resistant cases per 1000 were averted in Scenario 1, 2.7 (1.8–4.2) resistant cases per 1000 were averted in Scenario 2, 3.2 (2.2–5.1) resistant cases per 1000 were averted in Scenario 3, and 11.6 (7.8–17.3) resistant cases per 1000 were averted in a worst-case scenario in which TFRs increased to 80% by year 2030 (Supplementary Table 5).

**Table 2:** Malaria vaccine-averted burden by scenario, WHO Africa Region 2021–2030

Scenario	Cases per 1000 (Uncertainty Interval)	Resistant cases per 1000 (UI)	Deaths per 1000 (UI)
Baseline (no vaccine)	1409.1 (1143.6–1808.7)	3.8 (2.6–5.9)	3.8 (2.8–5.5)
	**Cases averted per 1000 (UI)**	**Resistant cases averted per 1000 (UI)**	**Deaths averted per 1000 (UI)**
VE1	235.7 (187.8–305.9)	0.6 (0.4–1.0)	0.6 (0.5–0.9)
VE2	313.9 (249.8–406.6)	0.8 (0.6–1.3)	0.9 (0.6–1.2)
VE3	384.7 (311.7–496.5)	1.0 (0.7–1.6)	1.1 (0.8–1.5)
VE1 with increasing resistance	235.7 (187.8–305.9)	10.4 (7.3–15.8)	0.6 (0.5–0.9)

Results of the baseline scenario (no vaccine) and three vaccine efficacy (VE) scenarios: 1) VE begins at 40% and drops 40 percentage points in year 5, 2) VE begins at 80% and drops 20 percentage points each year until reaching 0% in year five, and 3) VE remains 40% for the remainder of the study period. To show a worst-case scenario of ACT resistance, we modeled a scenario with the same VE as Scenario 1 but with delayed parasite clearance rates (DPCs) increasing to 80% by year 2030 (VE1 with Increasing Resistance). Results are cumulative over the 10-year study period and represent 42 African countries.

Annual results for outcomes averted per 1000 children are provided in Figs. 2–4. Supplementary Figures 3–5 show annual cumulative cases, resistant cases, and deaths per 1000 (not averted) for the baseline scenario and all VE scenarios. Supplementary Data 3 (model output data) and Supplementary Data 5–8 provide country results for all scenarios, including the baseline scenario without a vaccine and the worst-case scenario depicting increasing drug resistance. Country results for Scenario 1 are also presented in Supplementary Figures 6–8.

## Discussion

Our study, based on a mathematical model, indicates substantial malaria burden could be averted with a moderately effective vaccine regardless of waning time. Compared to a constant VE of 40% for 4 years dropping to 0% in year five (Scenario 1), the projected burden averted was greater when VE was higher right after vaccination (80%) and dropped 20 percentage points each year (Scenario 2) (Fig. 2). An initial spike followed by a waning period is consistent with VE trends of other vaccines and could be comparable to immunity after R21 Matrix-M vaccination; however, data from longitudinal studies of R21 Matrix-M are not yet available. Phase III trial results of RTS,S/AS01 indicated an average of approximately 40% efficacy over 48 months follow-up^42^; however, it is difficult to quantify exactly how this will change after 48 months, which is why we included Scenario 3 (VE remains constant at 40% for the entire period) for comparison. Cumulative cases averted per 1000 children in Scenario 3 surpassed those of Scenario 1 in 2025 and those of Scenario 2 in 2028 (Fig. 2), and the same trend was observed with resistant cases and deaths per 1000 averted (Figs. 3 and 4). The increasing resistance scenario in which DPCs rose to 80% in 10 years depicts a worst-case scenario (Fig. 5) of ACT resistance spread. Given modern treatments and management for malaria, it is unlikely that DPCs would rise to such a figure; however, this situation highlights the importance of vaccines among tools to mitigate burden caused by drug resistance.

In combination with other control strategies (e.g., chemoprevention, diagnostics, rapid access to treatment, and vector control products), an effective malaria vaccine could potentially reduce the disease burden even further than our projections indicate for several reasons. First, we assumed vaccines would only be administered annually to 1-year-olds over the 10-year period without a proposed catch-up campaign. Second, resistant cases averted in our study refer to detected and treated cases with parasitemia on day three of treatment. Many infections remain undetected, and individuals discontinue treatment over time. Third, we kept DPCs, TFRs, and CFRs constant over time when DPCs and TFRs are likely to increase with increasing drug resistance, which would consequently increase CFRs without adequate control interventions. Fourth, we did not apply different incidence rates for sensitive versus resistant cases due to data availability. The proportion of cases that are resistant in a population is likely to increase over time without effective management due to longer illness duration and recrudescence. Similarly, we did not apply different CFRs for sensitive versus resistant cases. Mortality averted may be greater than our estimates project if CFRs for resistant cases are higher than for sensitive cases.

We assumed that the efficacy of other prevention measures remained constant; however, the World Malaria Report indicates use of vector control products has been declining. Compared to nearly 60% in 2017, only 47% of the population in malaria endemic countries in sub-Saharan Africa used a bed net in 2021^53^. Compared to over 10% in 2010, only 5.3% of the population at-risk in the WHO Africa Region used residual spraying in 2021^53^. If the use of vector control products continues to decrease, the proportion of health events averted attributable to an effective vaccine could increase. If an effective vaccine were to become widely implemented, however, people may stop using bed nets and indoor residual spraying, which could lead to an increase in malaria burden despite a vaccine. In contrast to bed nets and indoor residual spraying, the use of seasonal malaria chemoprevention among children living in areas of highly seasonal malaria transmission has steadily increased since 2012 in the 13 Sahel countries in which it was implemented^53^ (Supplementary Data 1). Although seasonal malaria chemoprevention can be effective at reducing malaria morbidity and mortality during the high transmission season^54^, it requires efficient microplanning and treatment adherence. A recent clinical trial also indicated that seasonal malaria chemoprevention in combination with RTS,S/AS01 vaccination was more effective at preventing malaria cases and deaths than either prevention alone^29^. A malaria vaccine could also have the effect of prolonging the efficacy of drugs used in chemoprevention by reducing the number of infections. Future modeling studies could help evaluate the impact of wide scale implementation of RTS,S/AS01 with seasonal chemoprevention.

While we used the most recent publicly available country-specific data, our analysis has some caveats. First, we used a one-year time step, thus, our model did not mimic the multi-dose schedule of current malaria vaccines nor realistic immunity waning, which occurs gradually not in a stepwise fashion, limiting the ability to assess the impact of incomplete vaccination schedules. Second, the rate at which VE wanes depends on many factors, including the number and timing of doses, pathogen replication time and rate of exposure, and the effect on antibodies as well as B and T cell responses in the human host^55^. Deriving parameters for all of these factors was not possible due to limited data and lack of understanding of how these different factors interact. Our model combines these different factors into a single measure of VE and we use different scenarios to assess the potential range of VE over time. Future analyses on the impact of vaccines on drug-resistance should include increased complexity as data and/or understanding become more readily available.

Third, in addition to temporal variation, VE varies significantly within and between countries due to geographical differences in ecological, parasite, and human host factors (e.g., endemicity level, vaccine coverage, and incomplete vaccination schedules)^55^. The highest VE is usually observed in the lowest transmission settings. However, as country-specific estimates from trial data were only available for seven of the 42 countries in our study, we assumed the same VE for all countries. Similarly, we used country-specific DTP3 coverage rates as a proxy for malaria vaccine coverage rates; however, DTP3 coverage is likely much higher than initial country rates for RTS,S/AS01 coverage will be. We chose DPCs as the most appropriate proxy for drug resistance in the main analysis instead of molecular markers of *Pfkelch13* or TFRs, which are also reported in the Malaria Threat Map. The prevalence of *Pfkelch13* mutations and treatment failure rates likely overestimate resistance. *Pfkelch13* mutations can be, but are not always, associated with artemisinin partial resistance^6^ and were missing for several countries. In addition to resistance, treatment failure may be caused by poor compliance, inappropriate drug choice, or sub-therapeutic dosing^56^. Determining an appropriate proxy and accurate range for drug resistance is difficult as sample sizes are small and therapeutic efficacy studies tend to take place in hospitals and cities, which are not representative of the rest of a country. Estimates of drug-resistant cases calculated using TFRs in the sensitivity analysis provide an upper bound in a situation in which resistance remains constant over time (Scenarios 1–3). In both the main analysis and sensitivity analysis, the “worst-case” scenario provides an extreme upper bound in a situation where drug resistance increases rapidly over 10 years.

Fourth, a large number of therapeutic efficacy studies from the Malaria Threat Map had sample sizes less than 30 (we only included studies with 30 or more samples), and studies from African countries had an average of only 75 samples per study. Therapeutic efficacy studies are more likely to occur in high endemicity areas with adequate health system infrastructure. We imputed values for countries with missing treatment received and delayed parasite clearance rates, though this may have biased results. We did not account for increased partial immunity after an infection; however, we expect that the observed malaria incidence rates would reflect such dynamics. We also did not account for herd immunity. We did not apply different incidence or mortality rates for sensitive versus resistant cases. Future modeling studies that examine these differences should further clarify the relationship between vaccines and health burden caused by drug resistance.

Capturing the complexities of malaria transmission at scale and over time is very difficult mainly because of spatial heterogeneity due to variable degrees of stochasticity between low- and high-transmission areas and treatment rate. Additional examples include drug resistance, vaccine efficacy, and mortality rates dependent on drug type, severity of illness, and treatment duration, which can vary between and within countries. Models with more parameters that use smaller time-steps and aggregate at smaller geographical scales could provide more precise estimates. A review by Galactionova et al. highlights several finer scale models^34^, including a study by Hogan et al. that uses parameters at the first administrative unit level in addition to the country level in sub-Saharan Africa. The application of such detailed models to approximate public health impact for large regions may not be necessary or practical due to their extensive data needs for parameterization. Depending on study scope, simpler models may perform as well as more complex models^57^. The comparison of four models by Penny et al. found no statistical significance between the three dynamic models and the one static model^41^. While our projections for cases and deaths averted over a 10-year time horizon are higher than projections by Penny et al. over a 15-year time horizon, they are within the same order of magnitude. We determined the type of model, its specifications, and level of granularity given the large scope of this study (the entire WHO Africa Region). Accordingly, individual country results should be interpreted as aggregate estimates and with caution due to the many intra-country differences discussed.

While unknown factors on the evolution of drug resistance make it impossible to predict future health burden precisely, models using publicly available data produce useful aggregate projections at a macro scale. Our analysis could inform policymakers and vaccine developers on the urgency of an effective malaria vaccine. Development and implementation of an effective malaria vaccine should be accelerated to prevent the health and economic impacts of drug resistance, which will have a catastrophic impact when current therapies become ineffective^58^.

### Supplementary information

Description of Additional Supplementary Files

Supplementary Materials

Supplementary Data 1

Supplementary Data 2

Supplementary Data 3

Supplementary Data 4

Supplementary Data 5

Supplementary Data 6

Supplementary Data 7

Supplementary Data 8

Reporting Summary

## Data Availability

Source data used for results and figures are publicly available and accessible on GitHub^52^ (https://github.com/CDDEP-DC/Malaria_Vaccine-Averted_Burden) and in Supplementary Data 3. Additional data questions can be sent to the corresponding author. Original data sources are cited in Supplementary Table 1, Supplementary References, and in the Code Book sheet of Supplementary Data 2 (model input data).

## Code availability

All code is publicly available on GitHub^52^. Data were processed using Microsoft Office Excel 16.51, Stata 16, and Python 3.8. The final analyses and MC simulations were conducted in Python 3.8 with visualizations generated in RStudio 1.3.

## References

[1] World Health Organization. *Malaria Fact Sheet*. https://www.who.int/news-room/fact-sheets/detail/malaria (2022).

[2] World Health Organization. *World Malaria Report 2022*. https://www.who.int/publications-detail-redirect/9789240064898 (2022).

[3] World Health Organization (WHO). *World Malaria Report 2020: 20 Years of Global Progress and Challenges* (2020).

[4] Ndwiga L (2021). A review of the frequencies of Plasmodium falciparum Kelch 13 artemisinin resistance mutations in Africa. Int. J. Parasitol.: Drugs Drug Resist..

[5] White NJ (2021). Emergence of artemisinin-resistant Plasmodium falciparum in East Africa. N. Engl. J. Med..

[6] Uwimana A (2021). Association of Plasmodium falciparum kelch13 R561H genotypes with delayed parasite clearance in Rwanda: an open-label, single-arm, multicentre, therapeutic efficacy study. Lancet Infect. Dis..

[7] Uwimana A (2020). Emergence and clonal expansion of in vitro artemisinin-resistant Plasmodium falciparum kelch13 R561H mutant parasites in Rwanda. Nat. Med..

[8] Asua V (2021). Changing prevalence of potential mediators of aminoquinoline, antifolate, and artemisinin resistance across Uganda. J. Infect. Dis..

[9] World Health Organization (WHO). *Malaria Threats Map*. https://apps.who.int/malaria/maps/threats/ (2023).

[10] Amaratunga C (2012). Artemisinin-resistant Plasmodium falciparum in Pursat province, western Cambodia: a parasite clearance rate study. Lancet Infect. Dis..

[11] Slater HC, Griffin JT, Ghani AC, Okell LC (2016). Assessing the potential impact of artemisinin and partner drug resistance in sub-Saharan Africa. Malar. J..

[12] Krishna S, Kremsner PG (2013). Antidogmatic approaches to artemisinin resistance: reappraisal as treatment failure with artemisinin combination therapy. Trends Parasitol..

[13] Smith DL, Klein EY, McKenzie FE, Laxminarayan R (2010). Prospective strategies to delay the evolution of anti-malarial drug resistance: weighing the uncertainty. Malar. J..

[14] Vekemans J (2021). Leveraging vaccines to reduce antibiotic use and prevent antimicrobial resistance: a World Health Organization action framework. Clin. Infect. Dis..

[15] Nuwaha F (2001). The challenge of chloroquine-resistant malaria in sub-Saharan Africa. Health Policy Plan..

[16] Wellems TE, Plowe CV (2001). Chloroquine-resistant malaria. J. Infect. Dis..

[17] Trape JF (2001). The public health impact of chloroquine resistance in Africa. Am. J. Trop. Med. Hyg..

[18] Trape JF, Pison G (1998). Impact of chloroquine resistance on malaria mortality. C. R. Acad. Sci. III.

[19] Lubell Y (2014). Artemisinin resistance – modelling the potential human and economic costs. Malar. J..

[20] Lewnard JA, Lo NC, Arinaminpathy N, Frost I, Laxminarayan R (2020). Childhood vaccines and antibiotic use in low- and middle-income countries. Nature.

[21] Klein E, Schueller E, Tseng KK, Nandi A (2020). The impact of influenza vaccination on antibiotic use in the United States, 2010-2017. Open Forum Infect. Dis..

[22] Schueller E, Nandi A, Joshi J, Laxminarayan R, Klein EY (2021). Associations between private vaccine and antimicrobial consumption across Indian states, 2009–2017. Ann. N.Y. Acad. Sci..

[23] Andrejko K, Ratnasiri B, Hausdorff WP, Laxminarayan R, Lewnard JA (2021). Antimicrobial resistance in paediatric Streptococcus pneumoniae isolates amid global implementation of pneumococcal conjugate vaccines: a systematic review and meta-regression analysis. Lancet Microbe.

[24] Heymann DL, Kieny M-P, Laxminarayan R (2022). Adding to the mantra: vaccines prevent illness and death, and preserve existing antibiotics. Lancet Infect. Dis..

[25] Lewnard JA, Rogawski McQuade ET, Platts-Mills JA, Kotloff KL, Laxminarayan R (2020). Incidence and etiology of clinically-attended, antibiotic-treated diarrhea among children under five years of age in low- and middle-income countries: evidence from the Global Enteric Multicenter Study. PLOS Negl. Trop. Dis..

[26] Birger, R. et al. Estimating the effect of vaccination on antimicrobial-resistant typhoid fever in 73 countries supported by Gavi: a mathematical modelling study. *Lancet Infect. Dis.* **22**, 679–691 (2022). 10.1016/S1473-3099(21)00627-7. 10.1016/S1473-3099(21)00627-7 PMC9021026 35123673

[27] Fu H, Lewnard JA, Frost I, Laxminarayan R, Arinaminpathy N (2021). Modelling the global burden of drug-resistant tuberculosis avertable by a post-exposure vaccine. Nat. Commun..

[28] Centers for Disease Control and Prevention. *Barriers to Developing a Malaria Vaccine*. https://www.cdc.gov/malaria/malaria_worldwide/reduction/vaccine.html#:~:text=The%20development%20of%20a%20malaria,immune%20response%20to%20malaria%20infection. (2021).

[29] Chandramohan D (2021). Seasonal malaria vaccination with or without seasonal malaria chemoprevention. N. Engl. J. Med..

[30] World Health Organization. *Full Evidence Report on the RTS,S/AS01 Malaria Vaccine*. http://terrance.who.int/mediacentre/data/malaria/documents/mpag-october2021-session5-rtss-malaria-vaccine.pdf?sfvrsn=9507a63b_10 (2021).

[31] Datoo MS (2021). Efficacy of a low-dose candidate malaria vaccine, R21 in adjuvant Matrix-M, with seasonal administration to children in Burkina Faso: a randomised controlled trial. Lancet.

[32] Didierlaurent AM (2017). Adjuvant system AS01: helping to overcome the challenges of modern vaccines. Expert Rev. Vaccines.

[33] Datoo, M. S. et al. Efficacy and immunogenicity of R21/Matrix-M vaccine against clinical malaria after 2 years’ follow-up in children in Burkina Faso: a phase 1/2b randomised controlled trial. *Lancet Infect. Dis.*10.1016/S1473-3099(22)00442-X (2022). 10.1016/S1473-3099(22)00442-X 36087586

[34] Galactionova K, Smith TA, Penny MA (2021). Insights from modelling malaria vaccines for policy decisions: the focus on RTS,S. Malar. J..

[35] Struchiner CJ, Halloran ME, Spielman A (1989). Modeling malaria vaccines. I: New uses for old ideas. Math. Biosci..

[36] Halloran ME, Struchiner CJ, Spielman A (1989). Modeling malaria vaccines. II: Population effects of stage-specific malaria vaccines dependent on natural boosting. Math. Biosci..

[37] McCarthy KA, Wenger EA, Huynh GH, Eckhoff PA (2015). Calibration of an intrahost malaria model and parameter ensemble evaluation of a pre-erythrocytic vaccine. Malar. J..

[38] Griffin JT, Ferguson NM, Ghani AC (2014). Estimates of the changing age-burden of Plasmodium falciparum malaria disease in sub-Saharan Africa. Nat. Commun..

[39] Smith T (2012). Ensemble modeling of the likely public health impact of a pre-erythrocytic malaria vaccine. PLOS Med..

[40] Sauboin C, Van Bellinghen L-A, Van De Velde N, Van Vlaenderen I (2019). Economic impact of introducing the RTS,S malaria vaccine: cost-effectiveness and budget impact analysis in 41 countries. MDM Policy Pract..

[41] Penny MA (2016). Public health impact and cost-effectiveness of the RTS,S/AS01 malaria vaccine: a systematic comparison of predictions from four mathematical models. Lancet.

[42] RTS,S Clinical Trials Partnership. (2015). Efficacy and safety of RTS,S/AS01 malaria vaccine with or without a booster dose in infants and children in Africa: final results of a phase 3, individually randomised, controlled trial. Lancet.

[43] United Nations. *World Population Prospects 2019.* https://population.un.org/wpp/Download/Standard/Population/ (2020).

[44] Global Health Observatory. *Diphtheria Tetanus Toxoid and Pertussis (DTP3) Immunization Coverage among 1-year-olds (%)*. https://www.who.int/data/gho/data/indicators/indicator-details/GHO/diphtheria-tetanus-toxoid-and-pertussis-(dtp3)-immunization-coverage-among-1-year-olds-(-) (2021).

[45] Institute for Health Metrics and Evaluation. *Global Burden of Disease Results Tool*. https://vizhub.healthdata.org/gbd-results/ (2021).

[46] The Global Health Observatory. *Estimated Number of Malaria Cases*. https://www.who.int/data/gho/indicator-metadata-registry/imr-details/2971 (2021).

[47] The World Bank. *Children with Fever Receiving Antimalaria Drugs (% of Children under Age 5 with Fever)*. https://data.worldbank.org/indicator/SH.MLR.TRET.ZS (2018).

[48] World Health Organization. *Malaria: Artemisinin Partial Resistance*. https://www.who.int/news-room/questions-and-answers/item/artemisinin-resistance#:~:text=What%20is%20the%20definition%20of,following%20treatment%20with%20an%20ACT. (2022).

[49] World Health Organization. *Therapeutic Efficacy Test Protocol*. (2021) https://www.who.int/teams/global-malaria-programme/case-management/drug-efficacy-and-resistance/tools-for-monitoring-antimalarial-drug-efficacy.

[50] Global Health Observatory. *Estimated Malaria Deaths by Country*. https://www.who.int/data/gho/indicator-metadata-registry/imr-details/4650 (2022).

[51] Global Health Observatory. *Estimated Malaria Cases by Country*. https://www.who.int/data/gho/indicator-metadata-registry/imr-details/2971 (2022).

[52] Hamilton, A., Haghpanah, F., Lin, G. & Tulchinsky, A. *Malaria Vaccine Averted Burden GitHub Repository*. https://github.com/CDDEP-DC/Malaria_Vaccine-Averted_Burden (2023).

[53] World Health Organization. *World Malaria Report 2021*. https://www.who.int/publications/i/item/9789240040496 (2021).

[54] Baba E (2020). Effectiveness of seasonal malaria chemoprevention at scale in west and central Africa: an observational study. Lancet.

[55] Bell GJ (2021). Impacts of ecology, parasite antigenic variation, and human genetics on RTS,S/AS01e malaria vaccine efficacy. Curr. Epidemiol. Rep..

[56] White NJ (2009). Hyperparasitaemia and low dosing are an important source of anti-malarial drug resistance. Malar. J..

[57] White LJ (2009). The role of simple mathematical models in malaria elimination strategy design. Malar. J..

[58] OECD. *Stemming the Superbug Tide*. 10.1787/9789264307599-en (2018).

