# Supplementary material for: Modeling of malaria vaccine effectiveness on disease burden and drug resistance in 42 African countries
Source: Commun Med (Lond). 2023 Oct 13;3:144. doi: 10.1038/s43856-023-00373-y (PMC10576074; doi:10.1038/s43856-023-00373-y)
Supplement: Supplementary file 1 — Description of Additional Supplementary Files [file 43856_2023_373_MOESM1_ESM.docx]

**Description of Additional Supplementary Files**

**File Name:** Supplementary Data 1

**Description:** Country-specific parameters

**File Name:** Supplementary Data 2

**Description:** Model input data

**File Name:** Supplementary Data 3

**Description:** Model output data

**File Name:** Supplementary Data 4

**Description:** Baseline Cases, Resistant Cases, and Deaths per 1,000 Children by Country

**File Name:** Supplementary Data 5

**Description:** Scenario 1 Cases, Resistant Cases, and Deaths Averted per 1,000 Children by Country

**File Name:** Supplementary Data 6

**Description:** Scenario 2 Cases, Resistant Cases, and Deaths Averted per 1,000 Children by Country

**File Name:** Supplementary Data 7

**Description:** Scenario 3 Cases, Resistant Cases, and Deaths Averted per 1,000 Children by Country

**File Name:** Supplementary Data 8

**Description:** Resistant Cases Averted per 1,000 Children by Treatment Failure Rate (TFR) Scenario
