# Supplementary material for: Modeling of malaria vaccine effectiveness on disease burden and drug resistance in 42 African countries
Source: Commun Med (Lond). 2023 Oct 13;3:144. doi: 10.1038/s43856-023-00373-y (PMC10576074; doi:10.1038/s43856-023-00373-y)
Supplement: Supplementary file 2 — Supplementary Materials [file 43856_2023_373_MOESM2_ESM.pdf]

Supplement

Modeling of malaria vaccine effectiveness on disease burden and drug resistance in 42 African countries

Alisa Hamilton[1]
Fardad Haghpanah[1]
Mateusz Hasso-Agopsowicz[2]
Isabel Frost[2,3]
Gary Lin[1]
Emily Schueller[1]
Eili Klein[1,4]
Ramanan Laxminarayan[1,5,6]

[1] One Health Trust, Washington, DC, USA & New Delhi, India
[2] World Health Organization, Geneva, Switzerland
[3] Imperial College London, London, UK
[4] Johns Hopkins University, Department of Emergency Medicine, Baltimore, MD, USA
[5] Princeton University, Princeton, NJ, USA
[6] University of Washington, Seattle, WA, USA

Corresponding Author:

Ramanan Laxminarayan
One Health Trust
Washington, DC, USA
New Delhi, India
ramanan@onehealthtrust.org

Supplementary Table 1. Malaria Model Parameters

Parameter	Symbol	Definition	Value (Range)	Source
Target population	TP	Estimated number of children 1 years old	Country-specific (±10%) for each year 2021-2030	World Population Prospects 2019 (1)
Population by age group	W	Estimated number of children 1-4 years old	Country-specific for each year 2021-2030	World Population Prospects 2019 (1)
		Estimated number of children 5-9 years old		
Annual number of malaria cases	G	Estimated number of malaria cases among all ages	Country-specific	Global Health Observatory (2)
Annual number of malaria cases by age group	H	Estimated number of cases occurring among children 1-4 years old	Country Specific (Supplementary Equations 1)	Institutes of Health Metrics & Evaluation (3)
		Estimated number of cases occurring among children 5-9 years old		
		Estimated number of cases occurring among all ages		
At-Risk	k	Proportion of a country's population considered at-risk in malaria endemic countries	Country-specific	World Malaria Report 2022 (4)
Coverage	c	Proportion of one-year-olds receiving a vaccine	Country-specific DTP3 coverage	Global Health Observatory (5)
Efficacy	e	Proportion of vaccinated children protected from disease	Scenario 1: 40% (years 1-4), 0% (years 5+)	WHO expert consultation
			Scenario 2: 80% (year 1), 60% (year 2), 40% (year 3), 20% (year 4), 0% (years 5+)	
			Scenario 3: 40% all years	
Treatment received rate	r	Percentage of children under age five who were ill with fever in the last two weeks and received any appropriate (locally defined) anti-malarial drugs	Country-specific (±20%)	The World Bank (6): UNICEF, State of the World's Children, Childinfo, and Demographic and Health Surveys.
Delayed parasite clearance	d	Proportion of treated patients with parasitemia on day 3 of treatment. Estimated using studies of *P. falciparum* occurring in African countries	Country-specific (±20%)	Malaria Threat Map (7)
Treatment failure rate	f	Proportion of cases for which treatment fails. Estimated using studies of *P. falciparum* occurring in African countries	Country-specific (±20%)	Malaria Threat Map (7)
Case fatality rate	m	Proportion of cases that will result in death	0.256% (±20%) for low-transmission countries	World Malaria Report (4) Global Health Observatory (8,9)
			Estimated total deaths/estimated total cases (±20%) for high-transmission countries. Country-specific	

Supplementary Table 2. ACT Drug Names and Abbreviations

ACT Drug Name	Abbreviation	Average Percentage of patients with parasitemia on day 3 of treatment (Min–Max)*	Average Treatment Failure Rate (%) (Min–Max)*
Artemether-lumefantrine	AL	1.38 (0.00–23.10)	2.07 (0.00–42.60)
Artesunate + sulfadoxine-pyrimethamine	AS+SP	0.75 (0.00–9.20)	2.58 (0.00–25.90)
Artesunate-amodiaquine	AS-AQ	0.54 (0.00–22.60)	1.77 (0.00–22.60)
Artesunate-melfloquine	AS-MQ	18.79 (0.00–51.70)	4.76 (0.00–49.10)
Artesunate-pyronaridine	AS-PY	14.29 (0.00–46.70)	2.51 (0.00–18.00)
Dihydroartemisinin-piperaquine	DHA-PPQ	9.67 (0.00–74.80)	5.70 (0.00–68.10)

*****The proportion of patients with parasitemia on day three of treatment and the average treatment failure rate were calculated by taking the mean value for each drug using from data downloaded from the Malaria Threat Map from therapeutic efficacy studies of *P. falciparum* with over 30 samples (7).

Supplementary Table 3. Multivariate Regression Results for Treatment Received Rate (TRR) and Delayed Parasite Clearance (DPC) by GDP per Capita and Under 5 Mortality per 1,000

	Mean (SD)	95% Confidence Interval
Correlation between TRR and DPC	-0.052 (0.161)	(-0.352–0.247)
Intercept of TRR	-0.082 (0.161)	(-0.394–0.210)
Intercept of DPC	0.056 (0.125)	(-0.180–0.287)
Slope of GDP per Capita on TRR	-0.238 (0.179)	(-0.572–0.096)
Slope of GDP per Capita on DPC	-0.294 (0.176)	(-0.619–0.048)
Slope of Under 5 Mortality on TRR	0.380 (0.181)	(0.038–0.724)
Slope of Under 5 Mortality on DPC	-0.287 (0.178)	(-0.618–0.052)
Interaction of GDP per Capita and Under 5 Mortality on TRR	0.067 (0.204)	(-0.312–0.455)
Interaction of GDP per Capita and Under 5 Mortality on DPC	0.365 (0.210)	(-0.040–0.751)
Residual Standard Error for TRR	0.956 (0.114)	(0.755–1.173)
Residual Standard Error for DPC	0.923 (0.076)	(0.788–1.072)

*GDP per Capita was logged and standardized

**All values are on the same scale as standardized variables

Supplementary Table 4. Multivariate Regression Results for Treatment Received Rate (TRR) and Treatment Failure Rate (TFR) by GDP per Capita and Under 5 Mortality per 1,000

	**Mean (SD)**	**95% Confidence Interval**
Correlation between TRR and TFR	0.282 (0.141)	(0.020–0.539)
TRR Intercept	-0.003 (0.152)	(-0.283–0.287)
TFR Intercept	0.031 (0.123)	(-0.202–0.254)
Slope of GDP per Capita on TRR	-0.212 (0.162)	(-0.513–0.100)
Slope of GDP per Capita on TFR	-0.100 (0.178)	(-0.427–0.234)
Slope of Under 5 Mortality on TRR	0.334 (0.156)	(0.036–0.616)
Slope of Under 5 Mortality on TFR	-0.053 (0.166)	(-0.368–0.255)
Interaction of GDP per Capita and Under 5 Mortality on TRR	0.057 (0.165)	(-0.251–0.368)
Interaction of GDP per Capita and Under 5 Mortality on TFR	0.230 (0.185)	(-0.107–0.581)
Residual Standard Error for TRR	0.947 (0.113)	(0.753–1.162)
Residual Standard Error for TFR	0.985 (0.081)	(0.844–1.145)

GDP per Capita was logged and standardized

All values are on the same scale as standardized variables

Supplementary Table 5. Resistant Cases Averted using Delayed Parasite Clearance vs Treatment Failure as a Proxy for Drug Resistance, WHO Africa Region 2021-2030

Scenario	Resistant Cases per 1,000 using DPCs (Uncertainty Interval)	Resistant Cases per 1,000 using TFRs (UI)
Baseline (No Vaccine)	3.8 (2.6–5.9)	10.7 (7.1–16.7)
	**Resistant Cases Averted per 1,000 using DPCs (UI)**	**Resistant Cases Averted per 1,000 using TFRs (UI)**
VE1	0.6 (0.4–1.0)	2.0 (1.3–3.1)
VE2	0.8 (0.6–1.3)	2.7 (1.8–4.2)
VE3	1.0 (0.7–1.6)	3.2 (2.2–5.1)
VE1 with Increasing Resistance	10.4 (7.3–15.8)	11.6 (7.8–17.3)

Comparison of results from the main analysis using Delayed Parasite Clearance rates (DPCs) and results from the sensitivity analysis using Treatment Failure Rates (TFRs) as a proxy for drug resistance. Delayed parasite clearance is defined as parasitemia after three days of treatment (10). Treatment failure is defined as the development severe malaria symptoms within three days of treatment (early treatment failure), parasitemia in patients with signs of early treatment failure or fever between day four and the end of follow-up, or parasitemia between day seven and the end of follow-up (late treatment failure) (10). Results include the three vaccine efficacy (VE) scenarios and the worst-case scenario of increasing drug resistance. In the worst-case scenario, we modeled a scenario with the same VE as Scenario 1 but with DPCs (or TFRs) increasing to 80% by year 2030 (VE1 with Increasing Resistance). Results are cumulative over the 10-year study period and represent 42 African countries.

Supplementary Figure 1. Pairwise Ordinary Least Squares Regressions for GDP per Capita (G), Under 5 Mortality per 1,000 (U), Treatment Received Rate (R), and Delayed Parasite Clearance (F).

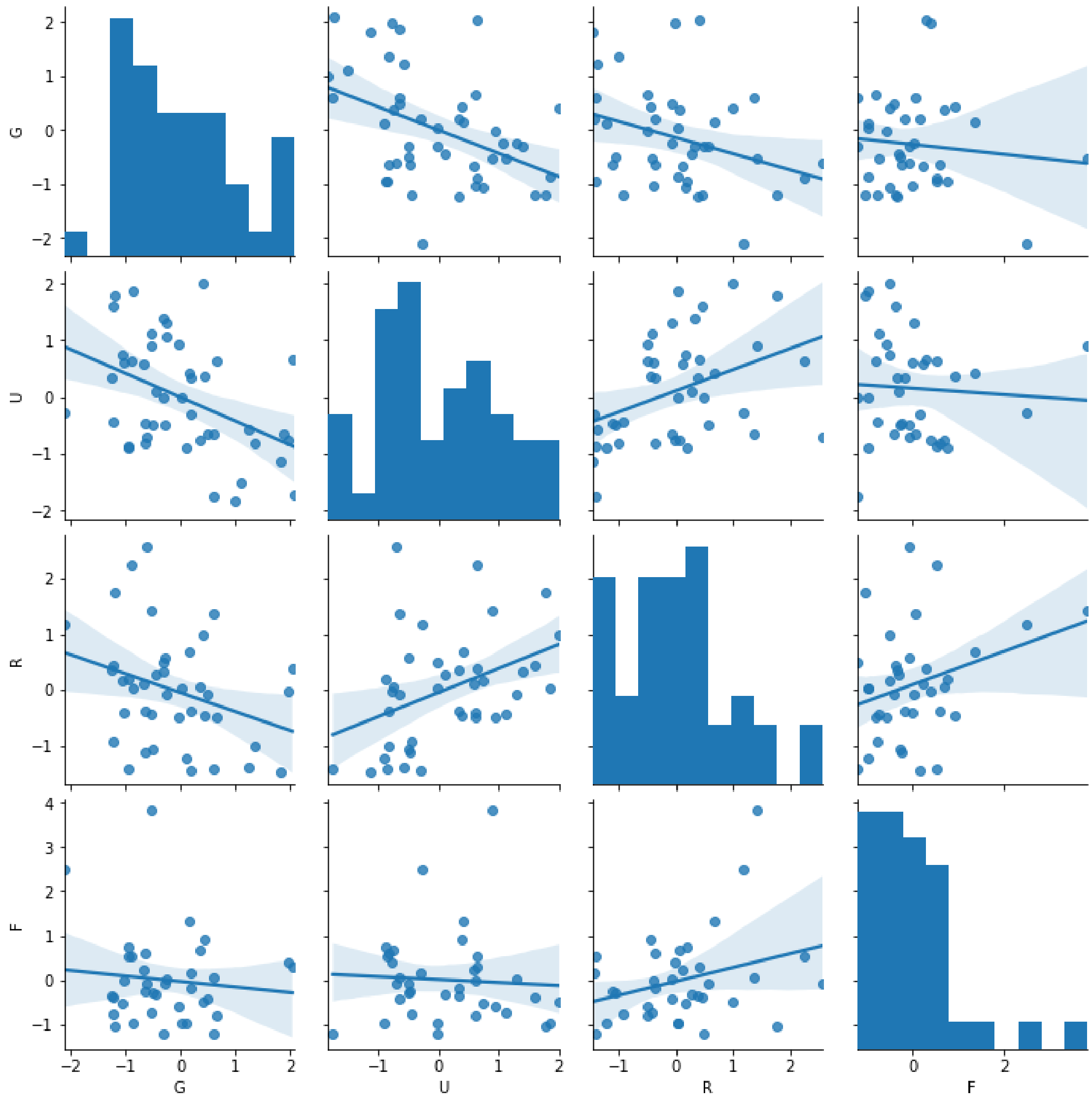

Treatment received rates (TRRs) were missing for Botswana, and Delayed Parasite Clearance rates (DPCs) were missing for Botswana, Eswatini, Guinea-Bissau, Namibia, South Africa, South Sudan, and Zimbabwe. Missing values were imputed using Bayesian multivariate regressing with GDP per capita and under-five mortality rates as predictor variables. These variables were selected as proxies for the strength of a country's economy and healthcare system, which can be reflective of malaria treatment and surveillance. Shaded regions represent 95% confidence intervals. Parameters were estimated using No-U-turn Hamiltonian Monte Carlo (11) and the Numpyro package version 0.10.1 in Python.

Supplementary Figure 2. Pairwise Ordinary Least Squares Regressions for GDP per Capita (G), Under 5 Mortality per 1,000 (U), Treatment Received Rate (R), and Treatment Failure Rate (F).

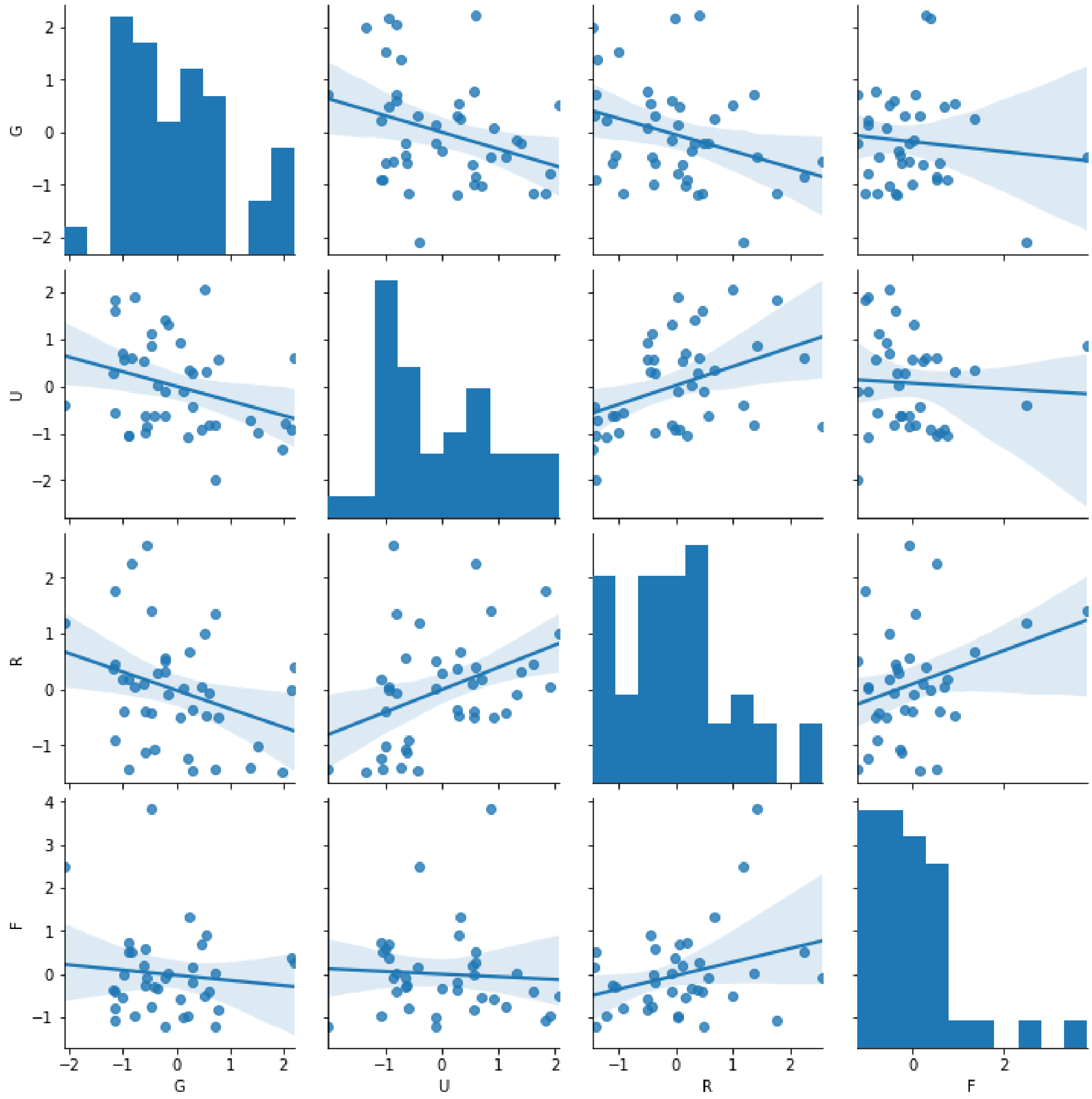

Treatment received rates (TRRs) were missing for Botswana, and treatment failure rates (TFRs) were missing for South Africa, Eswatini, Namibia, South Sudan, and Botswana. Missing values were imputed using Bayesian multivariate regressing with GDP per capita and under-five mortality rates as predictor variables. These variables were selected as proxies for the strength of a country's economy and healthcare system, which can be reflective of malaria treatment and surveillance. Shaded regions represent 95% confidence intervals. Parameters were estimated using No-U-turn Hamiltonian Monte Carlo (11) and the Numpyro package version 0.10.1 in Python.

Supplementary Figure 3. Cumulative Cases per 1,000 Children by Baseline and Vaccine Efficacy (VE) Scenario, WHO Africa Region 2021-2030.

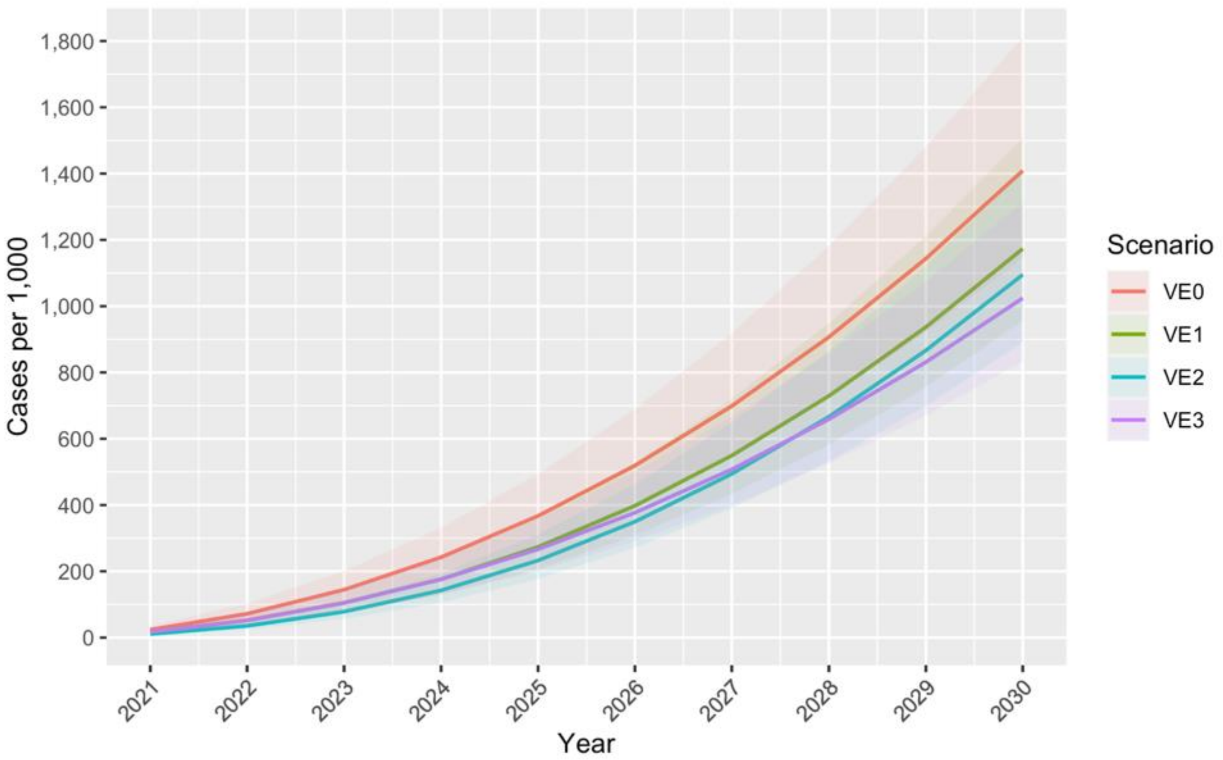

Shaded areas represent uncertainty intervals (UIs) calculated using Monte Carlo simulation with 1,000 iterations.

Supplementary Figure 4. Cumulative Resistant Cases per 1,000 Children by Baseline and Vaccine Efficacy (VE) Scenario, WHO Africa Region 2021-2030.

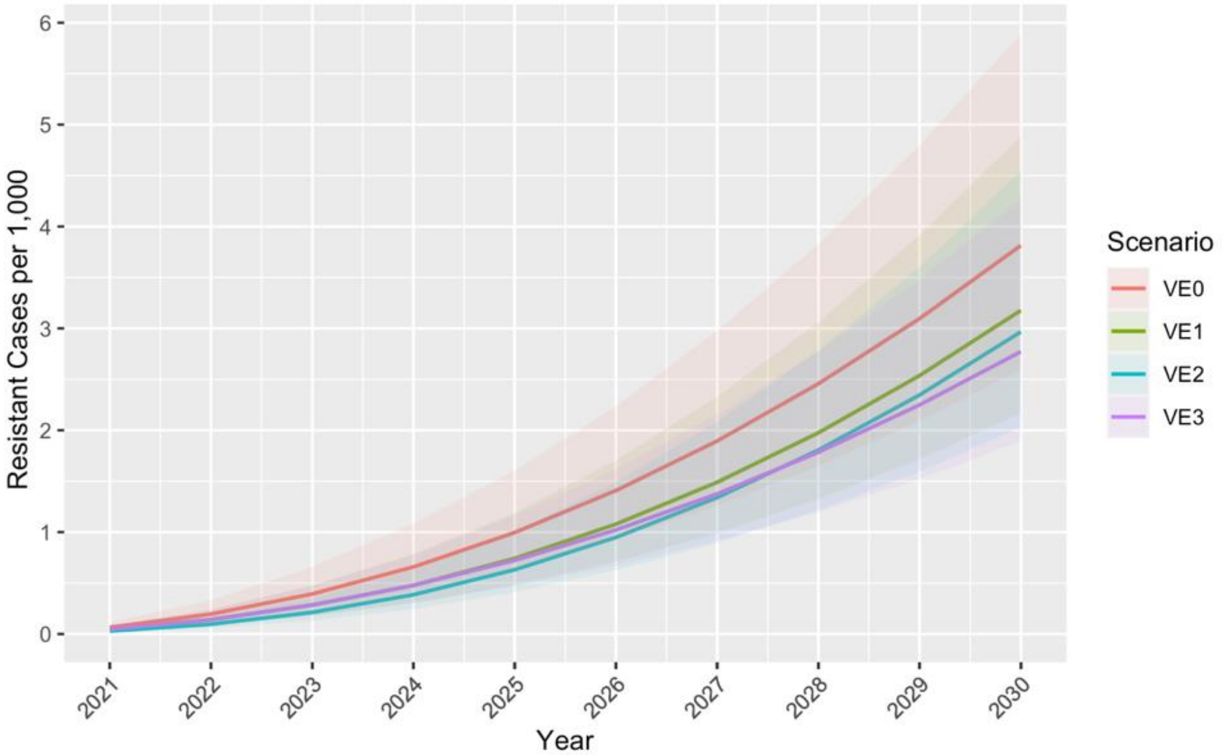

Shaded areas represent uncertainty intervals (UIs) calculated using Monte Carlo simulation with 1,000 iterations.

Supplementary Figure 5. Cumulative Deaths per 1,000 Children by Baseline and Vaccine Efficacy (VE) Scenario, WHO Africa Region 2021-2030.

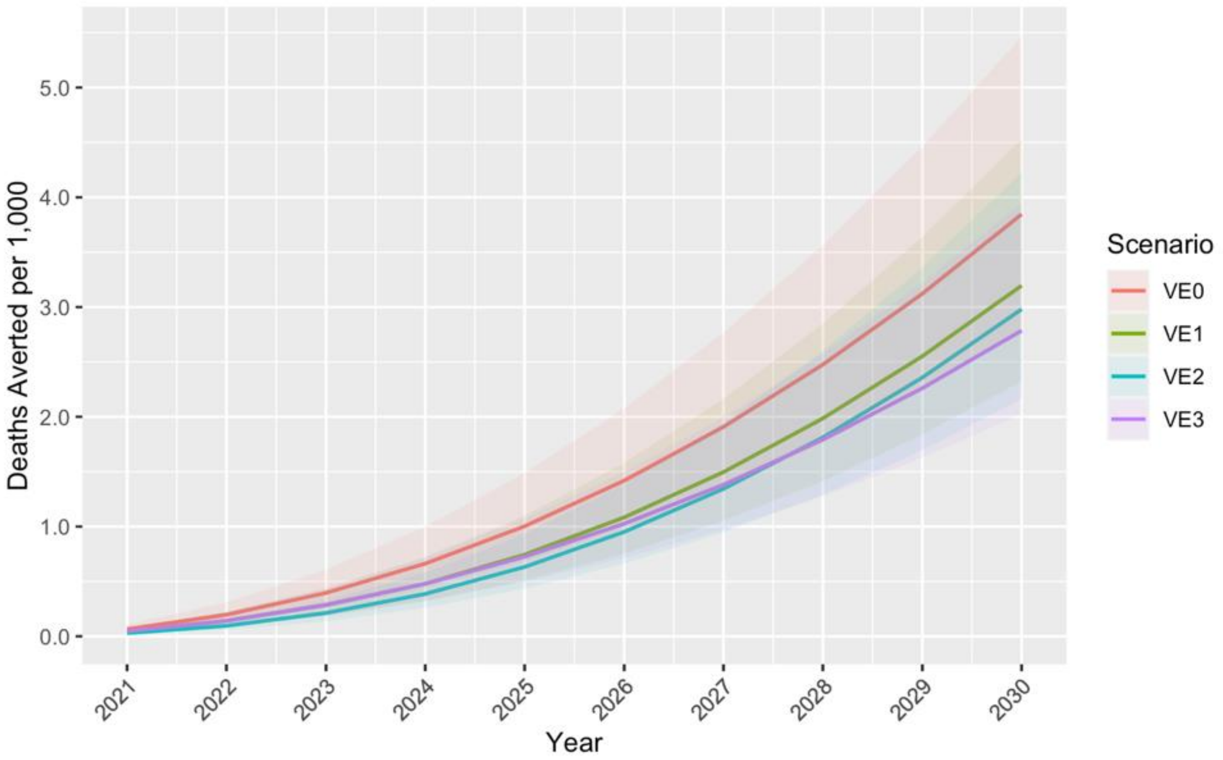

Shaded areas represent uncertainty intervals (UIs) calculated using Monte Carlo simulation with 1,000 iterations.

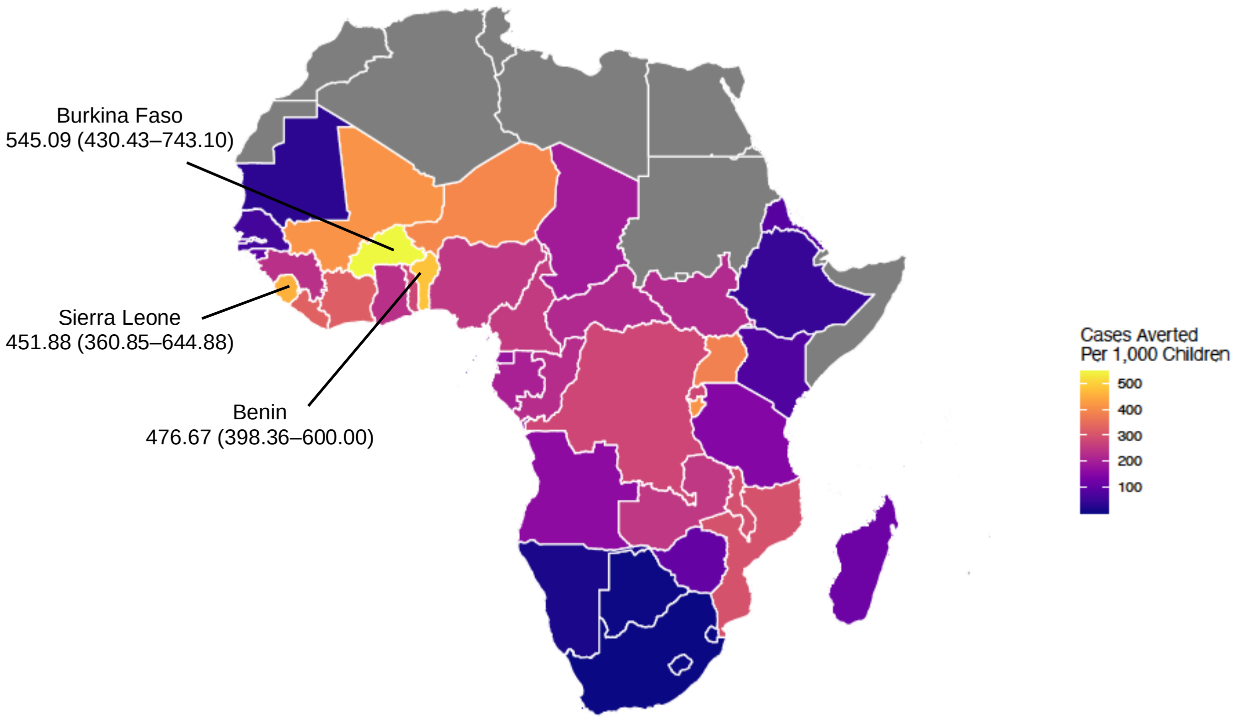

Supplementary Figure 6. Cumulative Cases Averted per 1,000 Children by Country (2021-2030). In a scenario with an effective vaccine with 40% efficacy and four years duration (Scenario 1), countries with the most cases averted per 1,000 population included Burkina Faso (545.09, Uncertainty Interval [UI] 430.43–743.10), Benin (476.67, UI 398.36–600.00), and Sierra Leone (451.88, UI 360.85–644.88). Maps were created in RStudio 1.3 using the ggplot2 package and shapefiles for Africa.

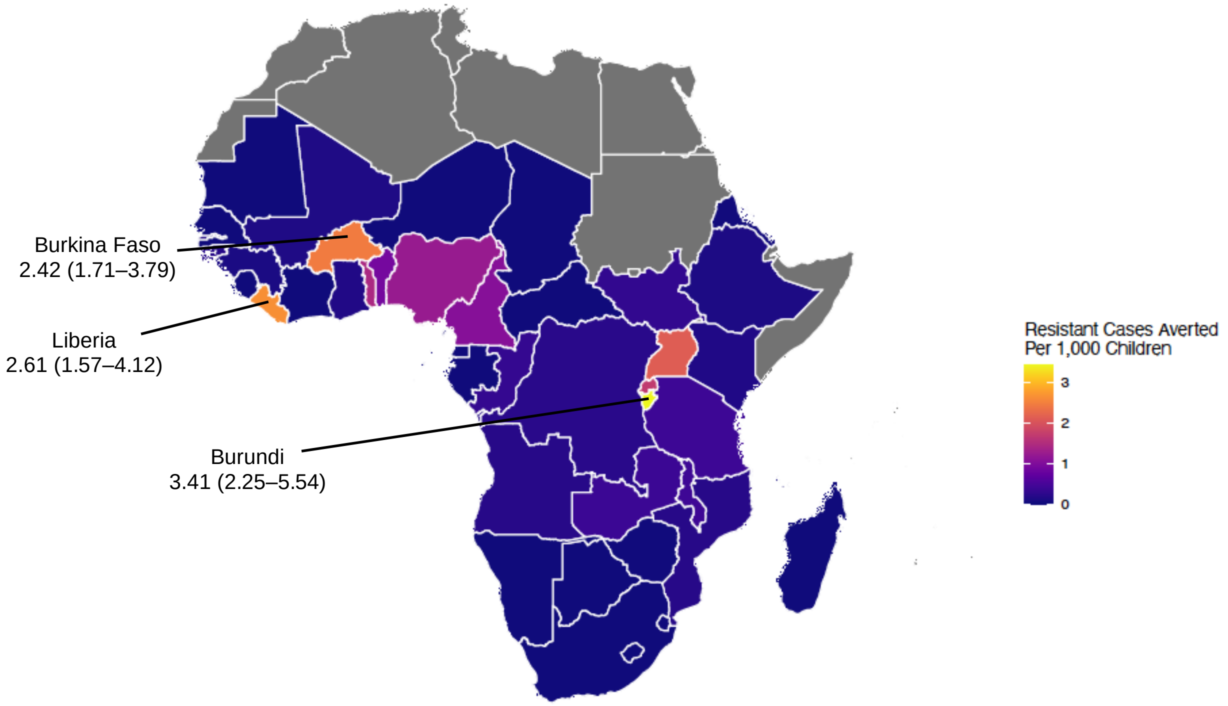

Supplementary Figure 7. Cumulative Resistant Cases Averted per 1,000 Children by Country (2021-2030). In a scenario with an effective vaccine with 40% efficacy and four years duration (Scenario 1), countries with the most resistant cases averted per 1,000 population included Burundi (3.41, Uncertainty Interval [UI] 2.25–5.54), Liberia (2.61, UI 1.57–4.12), and Burkina Faso (2.42, UI 1.71–3.79). Maps were created in RStudio 1.3 using the ggplot2 package and shapefiles for Africa.

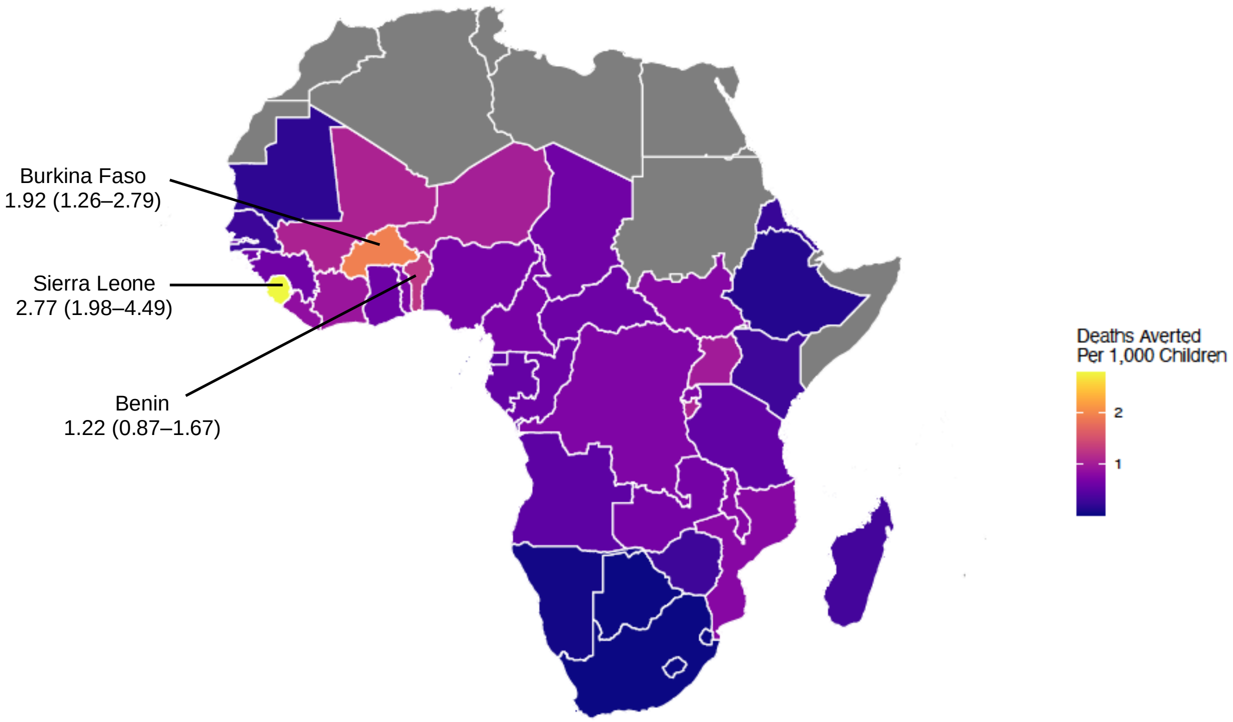

Supplementary Figure 8. Cumulative Deaths Averted per 1,000 Children by Country (2021-2030). In a scenario with an effective vaccine with 40% efficacy and four years duration (Scenario 1), countries with the most deaths averted per 1,000 population included Sierra Leone (2.77, Uncertainty Interval [UI] 1.98–4.49) Burkina Faso (1.92, UI 1.26–2.79), and Benin (1.22, UI 0.87–1.67). Maps were created in RStudio 1.3 using the ggplot2 package and shapefiles for Africa.

Supplementary Methods

Age group incidence rates were calculated using the following equation:

$$\beta_n = \frac{G * H_n}{W_n * H_t} * 1{,}000 \qquad (1)$$

...where β_n is the annual number of new cases per 1,000 population for age group (n), G is the estimated number of malaria cases among all ages reported by the Global Health Observatory, H_n is the estimated total number of malaria cases in age group (n) reported by The Institute for Health Metrics and Evaluation (IHME), H_t is the estimated total number of cases among all ages reported by IHME, and W_n is the estimated population of age group (n) reported by the United Nations World Population Prospects. Age groups 1-4 and 5-9 were reported by IHME. The incidence rate for children aged 5-9 was applied to children aged 10 in our study. Supplementary Table 4 provide country-specific values.

Supplementary References

1. United Nations. World Population Prospects 2019 [Internet]. Department of Economic and Social Affairs, United Nations Secretariat; 2020 [cited 2020 Apr 23]. Available from: https://population.un.org/wpp/Download/Standard/Population/

2. The Global Health Observatory. Estimated Number of Malaria Cases [Internet]. World Health Organization; 2021. Available from: https://www.who.int/data/gho/indicator-metadata-registry/imr-details/2971

3. Institute for Health Metrics and Evaluation. Global Burden of Disease Results Tool [Internet]. Institution for Health Metrics and Evaluation; 2021. Available from: https://vizhub.healthdata.org/gbd-results/

4. World Health Organization. World Malaria Report 2022 [Internet]. Geneva, Switzerland; 2022. Available from: https://www.who.int/publications-detail-redirect/9789240064898

5. Global Health Observatory. Diphtheria tetanus toxoid and pertussis (DTP3) immunization coverage among 1-year-olds (%) [Internet]. World Health Organization; 2021 [cited 2023 Mar 3]. Available from: https://www.who.int/data/gho/data/indicators/indicator-details/GHO/diphtheria-tetanus-toxoid-and-pertussis-(dtp3)-immunization-coverage-among-1-year-olds-(-)

6. The World Bank. Children with fever receiving antimalaria drugs (% of children under age 5 with fever) [Internet]. The World Bank; 2018. Available from: https://data.worldbank.org/indicator/SH.MLR.TRET.ZS

7. World Health Organization (WHO). Malaria Threats Map [Internet]. 2023 [cited 2023 Feb 20]. Available from: https://apps.who.int/malaria/maps/threats/

8. Global Health Observatory. Estimated Malaria Deaths by Country [Internet]. World Health Organization; 2022 Mar [cited 2022 Oct 5]. Available from: https://www.who.int/data/gho/indicator-metadata-registry/imr-details/4650

9. Global Health Observatory. Estimated Malaria Cases by Country [Internet]. World Health Organization; 2022 Mar [cited 2022 Oct 5]. Available from: https://www.who.int/data/gho/indicator-metadata-registry/imr-details/2971

10. World Health Organization. Therapeutic efficacy test protocol [Internet]. Geneva, Switzerland; [cited 2023 Jun 13]. Available from: https://www.who.int/teams/global-malaria-programme/case-management/drug-efficacy-and-resistance/tools-for-monitoring-antimalarial-drug-efficacy

11. Homan MD, Gelman A. The No-U-Turn Sampler: Adaptively Setting Path Lengths in Hamiltonian Monte Carlo. J Mach Learn Res. 2014 Jan;15(1):1593–623.
